# Enhancement of crystallization with nucleotide ligands identified by dye-ligand affinity chromatography

**DOI:** 10.1007/s10969-012-9124-8

**Published:** 2012-01-28

**Authors:** Heungbok Kim, Cecelia Webster, Justin K. M. Roberts, Juthamas Kositsawat, Li-Wei Hung, Thomas C. Terwilliger, Chang-Yub Kim

**Affiliations:** 1 Bioscience Division, Los Alamos National Laboratory, MS M888, Los Alamos, NM 87545 USA; 2 Department of Biochemistry, University of California, Riverside, CA 92521 USA; 3 School of Medicine, Loma Linda Medical School, Loma Linda, CA 92350 USA; 4 Physics Division, Los Alamos National Laboratory, MS D454, Los Alamos, NM 87545 USA

**Keywords:** Characterization of proteins based on ligands, Dye-ligand affinity chromatography, Enhancement of crystallization, Ligand aided crystallization, Ligand analysis, Nucleotide ligand

## Abstract

Ligands interacting with *Mycobacterium tuberculosis* recombinant proteins were identified through use of the ability of Cibacron Blue F3GA dye to interact with nucleoside/nucleotide binding proteins, and the effects of these ligands on crystallization were examined. Co-crystallization with ligands enhanced crystallization and enabled X-ray diffraction data to be collected to a resolution of at least 2.7 Å for 5 of 10 proteins tested. Additionally, clues about individual proteins’ functions were obtained from their interactions with each of a panel of ligands.

**Electronic supplementary material:**

The online version of this article (doi:10.1007/s10969-012-9124-8) contains supplementary material, which is available to authorized users.

## Introduction

Obtaining diffraction-quality crystals is a major bottleneck in determining macromolecular structures using X-ray crystallography [1, 2]. Various general approaches have been suggested for improving crystallization, including systematic screening of conditions [3]; seeding with small crystals [4]; cell-free expression [5]; screening multiple starting and ending amino acid positions for expression clones [6]; identifying domains by partial proteolysis [7, 8]; crystallizing complexes of small proteins [9, 10]; performing surface engineering [11]; using crystallization chaperones [12]; and supplementing with small-molecule ligands to increase crystallization [13] (See recent review of [14].

Ligand supplementation is particularly attractive as an option for improving crystallization in cases where ligand information for the target protein can be predicted from sequence-based functional annotation [15, 16]. If the right ligand is chosen, the effect on crystallization can be dramatic [17, 18]. In practice, the success rate of this approach is limited by the accuracy of ligand prediction for target proteins. In many cases, available annotations give little or no useful information on the ligand for target proteins. Estimates of error rates in annotation of gene function range from 5% to over 80% depending on the annotation methods and the database [19, 20], and this can lead to an incorrect choice of ligands. The unknown and hypothetical proteins that account for 25–60% of genome sequences give no indications of ligand [21–23]. As crystallization can be enhanced by addition of correctly selected ligand(s), accurate ligand information would be of substantial utility in crystallization.

After the fortuitous discovery of the binding of Cibacron Blue F3GA to enzymes led to the concept of using dyes for affinity chromatography [24], several triazine dyes were applied to enzyme purification as affinity resins. One of the most popular triazine dyes, Cibacron Blue F3GA, was found to interact with a variety of enzymes such as NAD- and NADP-dependent dehydrogenases, kinases, glucose oxidase, lysozyme, albumin, catalase, ovalbumin, as well as plasma proteins [25–31].

We have made use of the interaction of Cibacron Blue F3GA dye with nucleoside and nucleotide binding proteins to identify protein–ligand interactions [32]. Taking advantage of the binding of Cibacron Blue F3GA dye to a variety of cellular enzymes, we expanded the use of this dye from protein purification to high-throughput ligand identification of resin-bound proteins by selective elution with ligands [17, 32]. The premise of our approach is that a ligand that interacts specifically with a protein can release that protein from the dye-resin competitively. Crude cell extracts are applied to the dye-resin and the proteins eluted by each of a series of potential ligands are identified by two-dimensional electrophoresis and mass spectrometry [32]. Then the specificity of interactions is further tested by examining the elution of purified recombinant proteins from the dye-resin by individual ligands [17].

In this report, we demonstrate the general application of the F3GA dye-ligand affinity chromatography method to enhancing crystallization of 9 recombinant proteins chosen from a set of 26* Mycobacterium tuberculosis* (*Mtb*) targets that we previously found to interact with nucleoside/nucleotide ligands [32].

## Materials and methods

### Target selection, expression and purification of proteins

From our previous study with *Mtb* strain H37Rv cell extract in which we identified proteins interacting with nucleotide-ligands [32], 26 genes were selected and used in cloning. All genes in this study were amplified by PCR from a *Mtb* H37Rv genomic DNA, using Pfu polymerase (Stratagene), and the following primers: 5′-TACTTCCAATCCAATGCGAT G + N-terminal 20 nucleic acids coding region of target protein-3′ (forward) and 5′-TTATCCACTTCC AATGTTA + C-terminal 20 nucleic acids coding region of target protein-3′ (reverse). The underlined bases were to generate ligation-independent cloning (LIC) sites for the pMCSG7 vector [33]. The pMCSG7 vector was digested with the SspI restriction enzyme (Promega), and the amplified and purified PCR product and a singly digested pMCSG7 vector were treated with T4 DNA polymerase (Novagen). The 50 μl of *Escherichia coli* NovaBlue cells (Novagen) was transformed with the self-annealed PCR product and pMCSG7 vector. The insert of genes in plasmids was confirmed by DNA sequencing.

*E. coli* BL21(DE3) cells were used to express the cloned genes. Cells were grown at 37°C in LB medium (Sigma) containing 100 μg/ml ampicillin, induced with 1 mM IPTG when OD600 reached 1.0, and grown at 25°C overnight in a shaking incubator set at 250 rpm. The cells were harvested and stored at −80°C. The expression of each protein was checked by SDS-PAGE [34].

For purification of expressed proteins, frozen cells were thawed on ice and resuspended in lysis buffer (20 mM Tris–HCl pH 8.0, 200 mM NaCl, 1 mM PMSF, 1 mg/ml DNase, 1 mM MgCl_2_). Lysates were sonicated and then centrifuged with 3,000 g at 4°C for 30 min. The supernatant was filtered through a 0.45 μm pore membrane (Stericup, Millipore) and loaded on a Ni–NTA superflow affinity column (Qiagen). After being washed with buffer A (20 mM Tris–HCl pH 8.0, 200 mM NaCl), the target protein was eluted by buffer B (buffer A plus 500 mM imidazole). To remove the contaminants, eluted fractions were further purified on a Superdex-75 gel filtration column (GE Healthcare Inc.) using buffer C (10 mM Tris–HCl pH 8.0, 150 mM NaCl, and 1 mM DTT). A centrifugal concentrator (Millipore) was used to concentrate the pooled protein fractions to 5–15 mg/ml, as measured by Bradford reagent (Bio-Rad). Protein purity was confirmed by SDS-PAGE and densitometry. A summary of progress from solubility test to X-ray data collection is included in Supplementary Table 1.

### Nucleotide ligand analysis by dye-ligand affinity chromatography

To evaluate recombinant proteins for their specific ligand-binding properties, we followed a modified version of the protocol described by Kim et al*.* [17]. Briefly, individual purified proteins were diluted to 1–2 mg/ml in column buffer (CB; 50 mM potassium phosphate, pH 7.5, 1 mM MgCl_2_ and 2 mM DTT), and 100 μg protein was adsorbed to multiple 50 μl aliquots of F3GA resin (Bio-RAD) in 2 ml spin-columns (Costar, Fisher Scientific). Aliquots (F3GA resin + proteins) were vortexed for 1 h at 4°C for binding, followed by recovery of unbound protein (flow-through fraction) and washing of the resin (4 × 0.4 ml washes with CB); spin-columns were centrifuged for 30 s at 10,000 g to recover fractions and change solutions. Individual spin-columns containing resin-bound proteins were then incubated (as for protein binding) with 50 μl, 1 mM ligand in CB, and the eluate fraction was recovered by centrifugation. The ligands used in this experiment were NAD, NADH, NADP, NADPH, AMP, ADP, ATP, and GTP (FAD was used additionally for the conserved hypothetical protein). Aliquots of initial protein, flow-through, and eluate fractions were diluted with 1:1 volume ratio with 2 × SDS sample buffer, and 15 μl was loaded in equal proportion (equivalent to 1 μg input protein) on 10% SDS-PAGE. For quantitative evaluation of the interaction of each ligand with protein, stained gel-bands were scanned by densitometry (GS-800, Bio-RAD). We calculated a “densitometric trace” of each gel-band by integration of the absorbance of the stained band using the software, Quantity One 4.3.1. Each protein’s resin-bound portion was calculated by subtracting the portion of flow-through from the loaded protein. The protein portion eluted by each ligand was calculated as a percentage of the resin-bound protein. (Table 1 and Supplementary Table 2). We tested 33 of the 72 ligand–protein interactions examined in this work three times to estimate the uncertainty in our measurements of percentage bound to the dye column. The range of the percentage bound was less than ±15% from the mean in each case, and averaged ~10%.

### Crystallization and X-ray data collection of proteins without and with ligands

Proteins were crystallized without and with ligand(s) that had been identified with our dye-ligand interaction approach. For initial screening, hanging drops (1 μl protein–ligand solution + 1 μl reservoir solution) were set up in 24-well plates using crystal screen 1 and 2 (Hampton Research). To compare the effect of ligand(s) on crystallization under identical conditions, two or more drops that include one drop without ligand and other drop(s) with ligand(s) were set up on a cover-slip of each well depending on the number of identified ligands for each protein by dye-ligand affinity chromatography. If crystals were observed in multiple wells, we chose the well of best crystal based on size and morphology and optimized its crystallization condition, if possible, by fine-tuning each component until crystals with dimensions of at least 50 μm × 50 μm × 50 μm were obtained. For co-crystallization with ligand, each protein (0.2–0.5 mM in buffer C) was mixed with the identified ligand at a molar ratio of protein: ligand, 1:2.5 (and 1:5 to see the effect of enhancement of crystallization with more ligand, but no clear difference in enhancement of crystallization was observed depending on ligand concentration), and incubated on ice for 30 min prior to set-up crystallization. For X-ray data collection, a minimum of five protein crystals grown were selected based on size and morphology, harvested and flash-cooled in liquid N_2_, with the addition of 10–20% glycerol in the buffer as cryoprotectant.

Monochromatic datasets were collected at the beam lines 5.0.1 and 5.0.2 at the Advanced Light Source (ALS). The wavelengths used for each data collection and the estimated standard deviations (esd’s) of the wavelengths are listed in Table 2. All datasets were processed with the HKL2000 program suite [35]. Detailed data collection statics are listed in Table 2.

## Results and discussion

### Protein preparation and nucleotide ligand analysis by dye-ligand affinity chromatography

To determine whether the ligand(s) identified by F3GA dye-ligand affinity chromatography method can enhance crystallization, we selected 26 proteins from *Mtb* cell extracts for which the approach identified potential ligand interactions [32]. All 26 target genes were successfully cloned, and expression of each was confirmed. Solubility tests indicated that 10 of these were insoluble when expressed in *E. coli*, and these were not considered further. Crystal structures of three of the remaining 16 soluble proteins were already present in the Protein Data Bank (PDB, [36]; PDB codes listed in Supplementary Table 1) and these proteins were also not considered further. We purified the remaining 13 proteins.

The first step in our dye-ligand affinity chromatography experiment is normally evaluation of protein adsorption onto the Cibacron Blue F3GA dye resin. The buffer used in these experiments contains phosphate (50 mM), which we note might possibly compete weakly with dye binding and decrease weak or non-specific interaction of protein with dye resin. All proteins in this report had previously shown interactions with the F3GA resin, and binding to the resin was confirmed for all proteins (data not shown). For three of the 13 proteins, the ligand-specific elution analysis described below did not show interactions with the ligands we tested. These three (3-hydroxybutyryl-CoA dehydrogenase, probable fatty acid oxidation protein, and tryptophanyl-tRNA synthetase) were not considered further. Crystallization trials without and with ligand(s) identified in this analysis were set up for the remaining 10 proteins (Supplementary Table 1). Eight of these ten proteins (all but NAD(P) transhydrogenase and short-chain-type oxidoreductase) had previously been targeted by members of the Tuberculosis structural genomics consortium (TB SGC) for crystallization and structure determination, but various attempts to solve structures had failed (http://www.webtb.org).

**Fig. 1:**
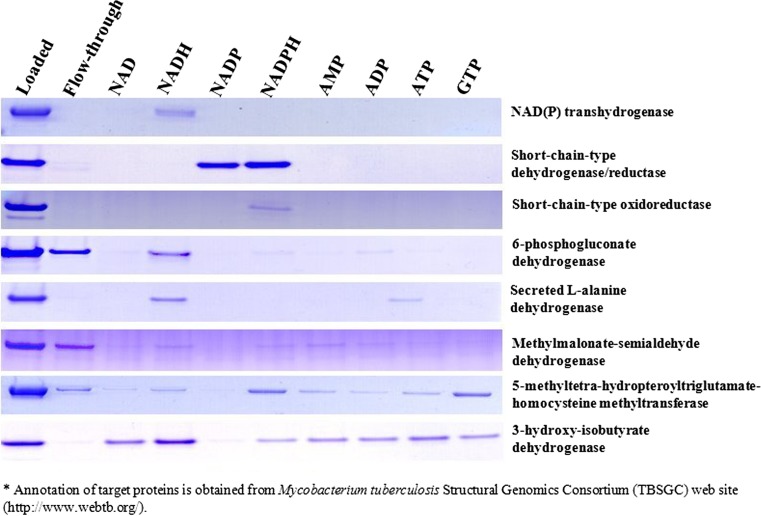
Nucleotide ligand interaction assay using *Mtb* recombinant proteins by dye-ligand affinity chromatography. The dye-ligand affinity chromatography results from SDS-PAGE show binding of eight target proteins to Cibacron Blue F3GA and subsequent selective elution in response to exposure to a sequence of eight different nucleotide ligands (NAD, NADH, NADP, NADPH, AMP, ADP, ATP, and GTP)

The enhancement of crystallization for one of these proteins (aldehyde dehydrogenase) with nucleoside ligands has been reported previously [17], and the experimental data for the remaining nine proteins are presented here. For all but one of these (the conserved hypothetical protein) addition of ligands improved crystallization (Supplementary Table 1). The eight proteins for which ligand addition improved crystallization are examined in more detail below. Figure 1 shows the application of the dye-ligand affinity chromatography approach to these eight *Mtb* proteins, testing interactions with eight common nucleotide ligands. The basis of this experiment is that the binding of ligand affects the binding of the protein to the resin. Initial binding of each protein to the resin was confirmed by the small relative amount in the flow-through fraction (lane 2) compared with the amount loaded (lane 1). For five of eight proteins, essentially all of the applied protein was bound to the resin. For the three others (6-phosphogluconate dehydrogenase, methylmalonate-semialdehyde dehydrogenase, and 5-methyltetra-hydropteroyl triglutamate-homocysteine methyl-transferase), unbound protein accounted for 14–37% of the loaded protein amount, respectively (Table 1). Ligands interacting with each of the eight proteins were identified by selective elution of the proteins. A relative measure of the interaction of each ligand with each protein could be obtained from the fraction of resin-bound protein that is eluted by that ligand (see “Materials and methods” and Supplementary Table 2), and is presented in Table 1.

**Table 1:** Estimation of retention of recombinant *Mtb* proteins on Cibacron Blue F3GA resin shown in Fig. 1

Proteins	Resin-bound portion of loaded protein (%)^a^	Eluted portion of bound protein (%)^b^							
		NAD	NADH	NADP	NADPH	AMP	ADP	ATP	GTP
NAD(P) transhydrogenase	100	0	24	0	0	0	0	0	0
Short-chain-type dehydrogenase/reductase	100	0	0	74	99	0	0	0	0
Short-chain-type oxidoreductase	100	0	0	0	15	0	0	0	0
6-phosphogluconate dehydrogenase	66	0	31	0	2	0	1	0	0
Secreted l-alanine dehydrogenase	100	0	55	0	0	0	0	11	0
Methylmalonate-semialdehyde dehydrogenase	63	0	13	0	5	8	0	0	0
5-methyltetra-hydropteroyltriglutamate-homocysteine methyltransferase	86	5	11	3	29	17	9	17	42
3-hydroxy-isobutyrate dehydrogenase	100	40	74	0	15	26	26	41	26

^a^The resin-bound portion of protein was estimated as a percentage of the total protein loaded by subtracting the flow-through portion from the loaded amount. Amounts of protein loaded and obtained in the flow-through were estimated from densitometry of stained SDS gels as in Fig. 1, as detailed in Supplementary Table 2

^b^The eluted portion of bound protein was calculated by dividing the amount of protein eluted with each ligand by the amount of resin bound protein

Of eight proteins shown in Fig. 1, five revealed very obvious interactions with ligands. The proteins annotated as NAD(P) transhydrogenase, short-chain-type oxidoreductase, 6-phosphogluconate dehydrogenase, and secreted l-alanine dehydrogenase showed elution dominated by one ligand (NADH or NADPH). The short-chain-type dehydrogenase/reductase was eluted with two ligands (NADP and NADPH). In the case of methylmalonate-semialdehyde dehydrogenase, three ligands showed interactions. In contrast, the proteins annotated as 5-methyltetra-hydropteroyl triglutamate-homocysteine methyltransferase and 3-hydroxy-isobutyrate dehydrogenase interacted with most of the ligands tested. Additionally, the conserved hypothetical protein showed interaction with FAD (Supplementary Tables 1 and 2).

Based on existing annotations, the eight proteins shown in Fig. 1 are likely all to be dehydrogenases or reductases, and they would be expected to interact with either NAD/NADH or NADP/NADPH. The ligand interactions indicated by our dye-elution analysis give clues as to the likely cofactors for several of these proteins. As NAD(P) transhydrogenase, 6-phosphogluconate dehydrogenase, and secreted l-alanine dehydrogenase are eluted most readily with NADH, we suggest that they may be NAD/NADH-dependent dehydrogenases. In contrast, short-chain-type dehydrogenase/reductase and short-chain-type oxidoreductase are eluted specifically by NADP and/or NADPH, and therefore may be NADP/NADPH-dependent enzymes. The proteins NAD(P) transhydrogenase, 6-phospho gluconate dehydrogenase, secreted l-alanine dehydrogenase, and short-chain-type oxidoreductase showed preferred interactions with the reduced form of cofactors (NADH or NADPH), suggesting that these enzymes may be involved in a reduction process.

During our work, structural and interaction data for secreted *Mtb* l-alanine dehydrogenase with NADH and other ligands was reported by Agren et al [37]. In agreement with our ligand interaction data, a strong interaction of this protein with NADH was confirmed (K_d_ = 8.2 μM and K_m_ = 10.7 μM) showing that this enzyme is likely to be involved more in reductive amination converting pyruvate and ammonia to l-alanine rather than reverse oxidative deamination. These observations were also supported by kinetic parameters of this enzyme: K_cat_ = 694 ± 33/s, K_m,pyruvate_ = 0.76 ± 0.05 mM for reductive amination versus K_cat_ = 126 ± 4/s, K_m,l-alanine_ = 15.64 ± 1.09 mM for oxidative deamination. This agreement with our observations suggests that protein–ligand interaction profiles obtained by dye-ligand affinity chromatography can produce not only information about ligand binding to a protein, but also useful functional information.

**Fig. 2:**
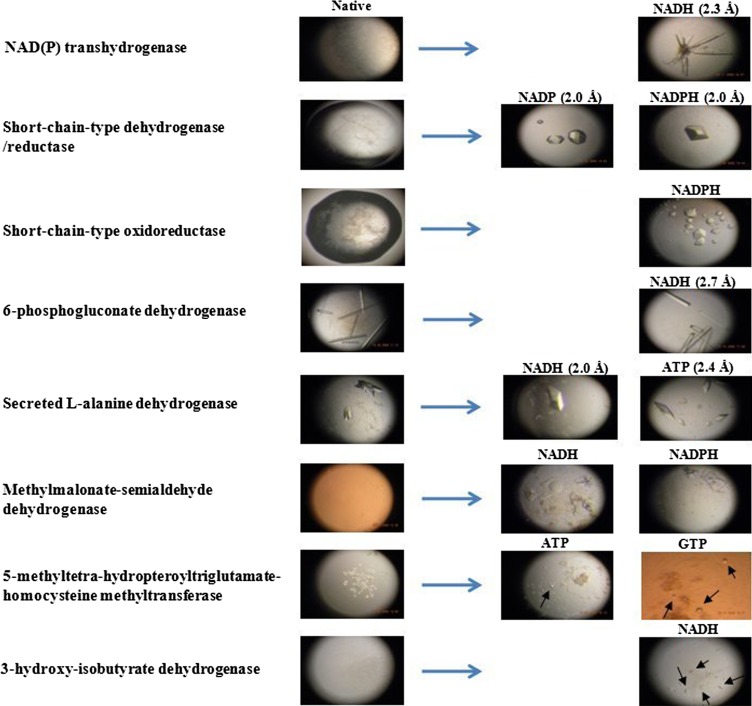
Ligand-enhanced crystallization of *Mtb* re*c*ombinant proteins. Proteins were crystallized in the absence (Native, left) or presence (right) of various nucleotide ligands, as indicated. Ligand selection was based on the results of the ligand interaction assay (Fig. 1 and Table 1). Enhanced crystallization of protein–ligand co–crystals, relative to native protein alone, was observed for all eight proteins, and allowed for structural resolution of four proteins (resolution of the collected diffraction data is indicated in *parentheses*)

Ligand(s) of a protein can be predicted from sequence-based annotation and from homologous protein’s structure solved with ligand. The challenges of ligand prediction by sequence-based annotation were mentioned above. Since all eight proteins used in this experiment are annotated in the category of dehydrogenases or reductases, we can predict all four nicotinamide nucleotides (NAD, NADH, NADP and NADPH) and can further suggest many nucleotides and nucleosides that are related to these as potential ligands, but it is hard to tell which one actually interacts with protein or improves crystallization until we screen each protein’s crystallization conditions with all of them. Similarly, when all the ligands bound to structures of proteins in the PDB with 30% or higher amino acid sequence identity for each of first five proteins in Fig. 1 are listed, many potential ligands are predicted for each protein. In contrast, ligands that showed clear interaction with protein by our method often enhanced crystallization as shown in Fig. 2, allowing us to make progress up to X-ray data collection as shown in Table 2.

**Table 2:** Statistics on X-ray data collection of four proteins, for which ligands information improved crystallization

Data collection	NAD(P) trans-hydrogenase	Short-chain-type dehydrogenase/reductase		6-phosphogluconate dehydrogenase	Secreted l-alanine dehydrogenase	
	NADH	NADP	NADPH	NADH	NADH	ATP
Space group	P2_1_	P2_1_2_1_2	P2_1_2_1_2	P42_1_2	R32	R32
Unit cell parameters (esd’s)						
a	65.144 (0.001)	160.450 (0.001)	159.395 (0.001)	108.280 (0.001)	88.154 (0.001)	89.047 (0.001)
b	151.841 (0.001)	159.952 (0.001)	160.801 (0.001)	108.280 (0.001)	88.154 (0.001)	89.047 (0.001)
c (Å)	76.306 (0.001)	86.189 (0.001)	172.583 (0.001)	144.938 (0.002)	291.390 (0.003)	290.981 (0.004)
α, β, γ (degree)	α = γ = 90, β = 114.38 (0.001)	α = β = γ = 90	α = β = γ = 90	α = β = γ = 90	α = β = 90, γ = 120	α = β = 90, γ = 120
Resolution limits^a^ (Å)	50.00–2.28 (2.32–2.28)	50.00–2.00 (2.03–2.00)	50.00–2.00 (2.03–2.00)	50.00–2.70 (2.75–2.70)	50.00–2.00 (2.03–2.00)	50.00–2.40 (2.43–2.40)
Wavelength (Å)	0.9774	1.0000	0.9202	1.0000	1.0000	1.0000
No. of Unique Reflections	59,257	148,901	298,159	24,403	29,900	17,977
No. of Total Reflections	209,793	1,088,722	2,214,370	167,780	177,183	106,091
R_merge_^b^ (%)	6.4 (28.0)	10.6 (93.7)	9.5 (63.3)	15.9 (78.5)	5.7 (58.9)	6.5 (34.9)
Completeness (%)	96.6 (85.0)	98.7 (97.9)	100.0 (100)	100.0 (99.7)	100.0 (99.9)	99.6 (99.4)
<*I*/σ(*I*)>	13.1 (3.6)	9.3 (2.0)	8.5 (2.8)	5.8 (2.0)	16.4 (2.6)	16.6 (4.4)
Redundancy	3.5 (3.0)	7.3 (6.4)	7.4 (7.4)	6.9 (6.2)	5.9 (5.6)	5.9 (5.8)

^a^Numbers in parentheses refer to the highest resolution shells

^b^ *R*_merge_ = Σ∣*I*_obs_ − *I*_avg_∣/Σ*I*_avg_

Inhibition studies of several enzymes such as phosphoglycerate kinase, phospho-glycerate mutase, cyclic 3′,5′-monophosphate dependent protein kinase, myosin subfragment 1, chloroplast coupling factor 1 using Cibacron Blue F3GA dye indicate that the dye inhibits enzyme activities by pre-occupation of the ligand binding site [38–41]. In the dye-ligand affinity chromatography approach, with the assumption that protein elution occurs by replacement of dye with nucleotide ligand at nucleotide binding sites, we may be able to design and test inactive enzyme variants by mutation of residues that are critical for ligand interaction once the enzyme-ligand interaction mode is elucidated by solving the enzyme structure bound to the ligand.

### Enhancement of crystallization by the ligands identified with dye-ligand elution

To examine the effects on crystallization of ligand(s) identified with our methods, nine *Mtb* proteins were crystallized without and with corresponding ligand(s) (Supplementary Table 1). Crystallization of six native proteins (without ligand) revealed no crystals in any condition and two (6-phosphogluconate dehydrogenase and secreted l-alanine dehydrogenase) generated relatively small crystals compared to proteins set up with ligands. In contrast, the co-crystallization of eight proteins (all but the conserved hypothetical protein) with ligands enhanced crystallization in various ways that we have never achieved with the native proteins (Fig. 2). Given the negative or poor crystallization results of native proteins relative to the results of protein with ligand(s) under identical crystallization conditions, we did not further pursue crystallization with native proteins by further screening of crystallization conditions or by protein modification. We categorize the enhancements by ligands in three groups: immediate formation of large new crystals (group 1), increases in crystal size (group 2), and the initiation of new crystals of small size (group 3). We found that of the proteins in group 1, NAD(P) transhydrogenase and short-chain-type dehydrogenase/reductase yielded new crystals diffracting to resolutions of 2.0–2.3 Å, whereas the crystals of short-chain-type oxidoreductase with NADPH diffracted but did not generate useful diffraction data and might need intensive screening of cryo-conditions. Of the proteins in group 2, larger crystals were obtained from 6-phosphogluconate dehydrogenase in the presence of NADH, yielding diffraction data to 2.7 Å resolution, and the crystals from secreted l-alanine dehydrogenase with NADH and ATP gave diffraction data to 2.0 Å and 2.4 Å resolution, respectively. When more than one ligand is identified, it may be useful to try tight-binding ligands first because they may yield better diffracting crystals [17]. The group 3 proteins methylmalonate-semialdehyde dehydrogenase, 5-methyltetra-hydropteroyl triglutamate-homocysteine methyltransferase, and 3-hydroxy-isobutyrate dehydrogenase yielded protein crystals with the ligands (NADH and NADPH, ATP and GTP, and NADH, respectively) that were confirmed by polarization and staining with dye, although those were too fragile or too small (5–10 μm in the largest dimensions) to harvest (the native proteins gave mere precipitation and no trace of crystals).

## Conclusion

We have demonstrated here the applicability of a novel method, dye-ligand affinity chromatography, to ligand screening of nine recombinant *Mtb* proteins.

A unique feature of this dye-ligand affinity chromatography procedure is the generation of information about ligand interactions, depending on each ligand’s ability to replace dye at the binding site of protein (Fig. 1). Depending on the character of the Cibacron Blue F3GA dye-protein interaction, the ligand-interaction profile obtained for a given protein can give clues to the protein’s function.

The advantages of this method over other ligand analyses, such as the thermal shift assay with ligands [13] and the ligand chips [42] are that (a) no protein modification is required, (b) no special instruments and skill are required, and (c) the potential for direct monitoring of ligand interaction by displacement of the dye from the binding site of the protein. These advantages may allow the development of a high-throughput ligand analysis system that can be operated in a cost- and time-effective manner.

In this report, the ligand(s) identified by this method demonstrated a 50% success rate (5 of 10 tested proteins, including aldehyde dehydrogenase, reported previously by Kim et al*.* [17]) for obtaining diffraction data by crystallization improvement. The structure of each protein for which we have collected data has been solved and is in the process of refinement (these structures will be reported elsewhere).

According to earlier reports about the proteins interacting with Cibacron Blue F3GA dye [25–31], most nucleoside/nucleotide-interacting proteins, or about half of all enzymes [43], can be candidates for ligand analysis using the dye-ligand affinity chromatography method. Based on our data, the method seems especially efficient for identifying ligand(s) if the target protein is annotated as an NAD/NADH-binding (and NADP/NADPH-binding) protein.

We suggest that a useful structural genomics approach will be to use our methods to identify ligands for target proteins early in the process and then to co-crystallize target proteins with the identified ligand(s), potentially yielding functional insights and improving crystal formation and diffraction data.

## Electronic supplementary material

Below is the link to the electronic supplementary material.

Supplementary material 1 (DOC 75 kb)

Supplementary material 2 (DOC 100 kb)

## Abbreviations

ADP: Adenosine-5′-diphosphate
AMP: Adenosine-5′-monophosphate
ATP: Adenosine-5′-triphosphate
DNA: Deoxyribonucleic acid
DTT: Dithiothreitol
FAD: Flavin-adenine dinucleotide
GTP: Guanosine-5′-triphosphate
IPTG: Isopropyl-β-d-thiogalactoside
NAD: Nicotinamide adenine dinucleotide
NADH: Nicotinamide adenine dinucleotide hydride
NADP: Nicotinamide adenine dinucleotide phosphate
NADPH: Nicotinamide adenine dinucleotide phosphate hydride
OD: Optical density
PCR: Polymerase chain reaction
PMSF: Phenylmethylsulfonyl fluoride
SDS-PAGE: Sodium dodecyl sulfate polyacrylamide gel electrophoresis
UTP: Uridine-5′-triphosphate
