# Supplementary material for: Enhancement of crystallization with nucleotide ligands identified by dye-ligand affinity chromatography
Source: J Struct Funct Genomics. 2012 Jan 28;13(2):71–9. doi: 10.1007/s10969-012-9124-8 (PMC3375012; doi:10.1007/s10969-012-9124-8)
Supplement: Supplementary file 1 — Supplementary material 1 (DOC 75 kb) [file 10969_2012_9124_MOESM1_ESM.doc]

**(Supplementary Table 1**) Summary of progress in structural genomics approach for 26 targets from solubility test up to x-ray data collection.

| # | *Mtb* Target proteins | | Solubility | | Purification | | Identification of ligand | Crystals | | | | Crystallization condition | | | X-ray data collection status | |
| --- | --- | --- | --- | --- | --- | --- | --- | --- | --- | --- | --- | --- | --- | --- | --- | --- |
|  |  |  |  |  |  |  |  | (no ligand) | | (with ligand) | |  |  |  |  |  |
| 1 | NAD[P] transhydrogenase | | Soluble | | Yes | | Yes | No | | Yes (NADH) | | 0.1 M Hepes-Na pH7.5, 1.5 M Lithium sulfate monohydrate | | | Yes^a^ | |
| 2 | Short-chain-type dehydrogenase/reductase | | Soluble | | Yes | | Yes | No | | Yes (NADP & NADPH) | | 0.1 M Sodium cacodylate pH6.8, 0.2 M Sodium acetate, 20 % PEG 8,000 | | | Yes^a^ | |
| 3 | Short-chain-type oxidoreductase | | Soluble | | Yes | | Yes | No | | Yes (NADPH) | | 0.4 M Potassium sodium tartrate tetrahydrate | | | No (screen of cryo-condition required) | |
| 4 | 6-phosphogluconate dehydrogenase | | Soluble | | Yes | | Yes | Yes | | Yes (NADH) | | 0.1 M Sodium acetate pH4.4, 2.1 M Sodium formate (with and without ligand) | | | Yes^a^ | |
| 5 | Secreted L-alanine dehydrogenase | | Soluble | | Yes | | Yes | Yes | | Yes (NADH & ATP) | | 2.0 M Sodium chloride, 10 % PEG 6,000 (with and without ligand) | | | Yes^a^ | |
| 6 | Methylmalonate-semialdehyde dehydrogenase | | Soluble | | Yes | | Yes | No | | Yes (NADH & NADPH) | | 0.5 M Sodium chloride, 0.01 M Magnesium chloride, 0.01 M Hexadecylammonium bromide | | | No (too fragile to harvest) | |
| 7 | 5-methyltetrahydropteroyltriglutamate-homocysteine methyltransferase | | Soluble | | Yes | | Yes | No | | Yes (ATP & GTP) | | 0.1 M Hepes pH7.5, 20 % Jaffamine M-600 | | | No (too small to harvest) | |
| 8 | 3-hydroxyisobutyrate dehydrogenase | | Soluble | | Yes | | Yes | No | | Yes (NADH) | | 0.2 M Magnesium formate dihydrate | | | No (too small to harvest) | |
| 9 | Conserved hypothetical protein | | Soluble | | Yes | | Yes | No | | No | |  | | |  | |
| 10 | Probable aldehyde dehydrogenase | | Soluble | | Yes | | Yes | Yes | | Yes (NAD, NADH & more) | | 0.1 M MES pH6.0, 1.26 M Ammonium sulfate (with and without ligand) | | | Yes^b^ | |
| 11 | 3-hydroxybutyryl-CoA dehydrogenase | | Soluble | | Yes | | No binding |  | |  | |  | | |  | |
| 12 | Probable fatty acid oxidation protein | | Soluble | | Yes | | No binding |  | |  | |  | | |  | |
| 13 | Tryptophanyl-tRNA synthetase | | Soluble | | Yes | | No binding |  | |  | |  | | |  | |
| 14 | Probable thioredoxin reductase | | Soluble | | (stopped) | |  |  | |  | |  | | | (PDB code 2A87: Structure solved without ligand)^c^ | |
| 15 | Nucleotide diphosphate kinase | | Soluble | | (stopped) | |  |  | |  | |  | | | (PDB code 1K44: Structure solved with GDP)^c^ | |
| 16 | Glutamine synthetase | | Soluble | | (stopped) | |  |  | |  | |  | | | (2WHI solved with purine analogue and MSO-P; 2WGS with purine analogue; 1HTO with AMP) ^c, d^ | |
| 17 | Possible ketoacyl reductase | | Insoluble | | No | |  |  | |  | |  | | |  | |
| 18 | Probable aldehyde dehydrogenase | | Insoluble | | No | |  |  | |  | |  | | |  | |
| 19 | probable aldehyde dehydrogenase | | Insoluble | | No | |  |  | |  | |  | | |  | |
| 20 | Phosphopyruvate hydratase | | Insoluble | | No | |  |  | |  | |  | | |  | |
| 21 | Probable acyl-[acyl-carrier-protein] desaturase | | Insoluble | | No | |  |  | |  | |  | | |  | |
| 22 | Acetyl-CoA acetyltransferase | | Insoluble | | No | |  |  | |  | |  | | |  | |
| 23 | Possible bifunctional enzyme riboflavin biosynthesis protein | | Insoluble | | No | |  |  | |  | |  | | |  | |
| 24 | Piperideine-6-carboxilic acid dehydrogenase | | Insoluble | | No | |  |  | |  | |  | | |  | |
| 25 | GDP-D-mannose 4,6 dehydratase | | Insoluble | | No | |  |  | |  | |  | | |  | |
| 26 | Probable inosine-5'-monophosphate dehydrogenase | | Insoluble | | No | |  |  | |  | |  | | |  | |
| ^a^The diffraction data was collected as shown in Table 2. | | | | | | | | | | | | | | | |  |
| ^b^The data has been reported in our previous report (17). | | | | | | | | | | | | | | | |  |
| ^c^The work was stopped because the structure was solved by others and deposited in PDB during our cloning process. | | | | | | | | | | | | | | | |  |
| ^d^Purine analogue, 1-[(3,4-dichlorophenyl)methyl]-3,7-dimethyl-8-morpholin-4-yl-purine-2,6-dione; MSO-P, L-methionine-S-sulfoximine phosphate | | | | | | | | | | | | | | | |  |
|  | |  | |  | |  | | |  | |  | |  |  | |  |
