# Supplementary material for: Enhancement of crystallization with nucleotide ligands identified by dye-ligand affinity chromatography
Source: J Struct Funct Genomics. 2012 Jan 28;13(2):71–9. doi: 10.1007/s10969-012-9124-8 (PMC3375012; doi:10.1007/s10969-012-9124-8)
Supplement: Supplementary file 2 — Supplementary material 2 (DOC 100 kb) [file 10969_2012_9124_MOESM2_ESM.doc]

**(Supplementary Table 2)** Densitometry and estimation of the bound protein portion relative to the loaded protein and the eluted portion by each ligand relative to the bound protein.

|  | | | | | | | | | |
| --- | --- | --- | --- | --- | --- | --- | --- | --- | --- |
| Proteins |  | Loaded | |  | (a) Flow-Through and Bound portions (relative to the Loaded protein) | | | | |
|  |  |  |  |  | Flow-Through | |  | Bound (calculated) | |
|  |  | Densitometric trace^a^ (OD x mm) | Converted to % |  | Densitometric trace^a^ (OD x mm) | % relative to loaded |  | Densitometric trace^b^ (OD x mm) | % relative to loaded |
|  |  |  |  |  |  |  |  |  |  |
| NAD(P) transhydrogenase |  | 0.591 | 100 |  | 0 | 0 |  | 0.591 | 100 |
| Short-chain-type dehydrogenase/reductase |  | 0.531 | 100 |  | 0 | 0 |  | 0.531 | 100 |
| Short-chain-type oxidoreductase |  | 0.697 | 100 |  | 0 | 0 |  | 0.697 | 100 |
| 6-phosphogluconate dehydrogenase |  | 0.855 | 100 |  | 0.288 | 34 |  | 0.567 | 66 |
| Secreted L-alanine dehysrogenase |  | 0.500 | 100 |  | 0 | 0 |  | 0.500 | 100 |
| Methylmalonate-semialdehyde dehydrogenase |  | 0.413 | 100 |  | 0.153 | 37 |  | 0.260 | 63 |
| 5-methyltetra-hydropteroyltriglutamate-homocysteine methyltransferase |  | 0.833 | 100 |  | 0.118 | 14 |  | 0.715 | 86 |
| 3-hydroxy-isobutyrate dehydrogenase |  | 0.404 | 100 |  | 0 | 0 |  | 0.404 | 100 |
| Conserved hypothetical protein |  | 0.363 | 100 |  | 0 | 0 |  | 0.363 | 100 |
|  |  |  |  |  |  |  |  |  |  |
| Proteins |  | (b) Eluted portions by each ligand (relative to the bound protein) | | | | | | | |
|  |  | NAD | |  | NADH | |  | NADP | |
|  |  | Densitometric trace^a^ (OD x mm) | % relative to bound |  | Densitometric trace^a^ (OD x mm) | % relative to bound |  | Densitometric trace^a^ (OD x mm) | % relative to bound |
|  |  |  |  |  |  |  |  |  |  |
| NAD(P) transhydrogenase |  | 0 | 0 |  | 0.144 | 24 |  | 0 | 0 |
| Short-chain-type dehydrogenase/reductase |  | 0 | 0 |  | 0 | 0 |  | 0.394 | 74 |
| Short-chain-type oxidoreductase |  | 0 | 0 |  | 0 | 0 |  | 0 | 0 |
| 6-phosphogluconate dehydrogenase |  | 0 | 0 |  | 0.178 | 31 |  | 0 | 0 |
| Secreted L-alanine dehysrogenase |  | 0 | 0 |  | 0.273 | 55 |  | 0 | 0 |
| Methylmalonate-semialdehyde dehydrogenase |  | 0 | 0 |  | 0.033 | 13 |  | 0 | 0 |
| 5-methyltetra-hydropteroyltriglutamate-homocysteine methyltransferase |  | 0.034 | 5 |  | 0.079 | 11 |  | 0.020 | 3 |
| 3-hydroxy-isobutyrate dehydrogenase |  | 0.162 | 40 |  | 0.297 | 74 |  | 0 | 0 |
| Conserved hypothetical protein |  | 0 | 0 |  | 0 | 0 |  | 0 | 0 |
|  |  |  |  |  |  |  |  |  |  |
| Proteins |  | (b) Eluted portions by each ligand (relative to the bound protein) | | | | | | | |
|  |  | NADPH | |  | AMP | |  | ADP | |
|  |  | Densitometric trace^a^ (OD x mm) | % relative to bound |  | Densitometric trace^a^ (OD x mm) | % relative to bound |  | Densitometric trace^a^ (OD x mm) | % relative to bound |
|  |  |  |  |  |  |  |  |  |  |
| NAD(P) transhydrogenase |  | 0 | 0 |  | 0 | 0 |  | 0 | 0 |
| Short-chain-type dehydrogenase/reductase |  | 0.525 | 99 |  | 0 | 0 |  | 0 | 0 |
| Short-chain-type oxidoreductase |  | 0.105 | 15 |  | 0 | 0 |  | 0 | 0 |
| 6-phosphogluconate dehydrogenase |  | 0.009 | 2 |  | 0 | 0 |  | 0.008 | 1 |
| Secreted L-alanine dehysrogenase |  | 0 | 0 |  | 0 | 0 |  | 0 | 0 |
| Methylmalonate-semialdehyde dehydrogenase |  | 0.013 | 5 |  | 0.021 | 8 |  | 0 | 0 |
| 5-methyltetra-hydropteroyltriglutamate-homocysteine methyltransferase |  | 0.206 | 29 |  | 0.118 | 17 |  | 0.061 | 9 |
| 3-hydroxy-isobutyrate dehydrogenase |  | 0.062 | 15 |  | 0.105 | 26 |  | 0.106 | 26 |
| Conserved hypothetical protein |  | 0 | 0 |  | 0 | 0 |  | 0 | 0 |
|  |  |  |  |  |  |  |  |  |  |
|  |  |  |  |  |  |  |  |  |  |
| Proteins |  | (b) Eluted portions by each ligand (relative to the bound protein) | | | | | | | |
|  |  | ATP | |  | GTP | |  | FAD | |
|  |  | Densitometric trace^a^ (OD x mm) | % relative to bound |  | Densitometric trace^a^ (OD x mm) | % relative to bound |  | Densitometric trace^a^ (OD x mm) | % relative to bound |
|  |  |  |  |  |  |  |  |  |  |
| NAD(P) transhydrogenase |  | 0 | 0 |  | 0 | 0 |  | NA^c^ | NA |
| Short-chain-type dehydrogenase/reductase |  | 0 | 0 |  | 0 | 0 |  | NA | NA |
| Short-chain-type oxidoreductase |  | 0 | 0 |  | 0 | 0 |  | NA | NA |
| 6-phosphogluconate dehydrogenase |  | 0 | 0 |  | 0 | 0 |  | NA | NA |
| Secreted L-alanine dehysrogenase |  | 0.055 | 11 |  | 0 | 0 |  | NA | NA |
| Methylmalonate-semialdehyde dehydrogenase |  | 0 | 0 |  | 0 | 0 |  | NA | NA |
| 5-methyltetra-hydropteroyltriglutamate-homocysteine methyltransferase |  | 0.122 | 17 |  | 0.298 | 42 |  | NA | NA |
| 3-hydroxy-isobutyrate dehydrogenase |  | 0.165 | 41 |  | 0.106 | 26 |  | NA | NA |
| Conserved hypothetical protein |  | 0 | 0 |  | 0 | 0 |  | 0.031 | 9 |
|  | | | | | |  |  |  |  |
| ^a^ The integrated staining estimated by densitometry (band intensity by optical density (OD) and band width by mm)  ^b^The calculated densitometric trace by subtracting densitometric trace of the flow-through from densitometric trace of the loaded | | | | | | |  |  |  |
| ^c^ Data not available |  |  |  |  |  |  |  |  |  |
